# Preliminary Testing of the English Version of the Adolescent Screener for Intelligence and Learning Disabilities (SCIL) Among Adolescents in Nigeria

**DOI:** 10.1111/jir.70136

**Published:** 2026-07-02

**Authors:** Eziafakaku Uchechukwu Nwokolo, Glynis H. Murphy, Anne‐Marie Mensink, Xavier Moonen, Peter E. Langdon

**Affiliations:** ^1^ Tizard Centre University of Kent Canterbury UK; ^2^ Norfolk Community Health and Care NHS Trust Norwich UK; ^3^ Department of Child Development and Education, Faculty of Social and Behavioural Sciences University of Amsterdam Amsterdam the Netherlands; ^4^ Intellectual Disabilities Research Institute (IDRIS) University of Birmingham Birmingham UK; ^5^ Birmingham Community Healthcare NHS Foundation Trust Birmingham UK; ^6^ Herefordshire and Worcestershire Health and Care NHS Trust Worcester UK

**Keywords:** adolescent, Africa, diagnosis, intellectual disabilities, Nigeria, screening

## Abstract

**Background:**

There are few screening tools for intellectual disabilities that have been developed and used within Africa. This study aimed to examine the psychometric properties of the English version of the adolescent Screener for Intelligence and Learning Disabilities (SCIL) when used with Nigerian adolescents and young people.

**Method:**

Two hundred nine adolescents and young people (aged 11–26 years) completed the SCIL and took part in an assessment of their level of general intellectual functioning and adaptive behaviour. Initially, principal components analysis was used to determine whether SCIL items should be retained or removed. Discriminative and convergent validity were then examined, along with the sensitivity, specificity, positive predictive (PPV) and negative predictive (NPV) values, using adjusted and nonadjusted diagnostic criteria for intellectual disability. The diagnostic criteria were adjusted because Western intelligence tests were not developed for use within Africa, and their use without adjustment remains contentious.

**Results:**

All SCIL items were retained. The SCIL had good internal consistency, discriminative and convergent validity. Using adjusted diagnostic criteria, a SCIL cutoff score of 10 revealed sensitivity = 0.66, specificity = 1, PPV = 1 and NPV = 0.83 for identifying those with an intellectual disability. AUC was 0.91. Unadjusted diagnostic criteria and the original SCIL cutoff score of 15, revealed sensitivity = 0.67, specificity = 0.98, PPV = 0.96 and NPV = 0.82 with an AUC of 0.96.

**Conclusions:**

The SCIL has good psychometric properties when used with Nigerian adolescents. Further factor analytic work is needed.

Social factors such as parental knowledge and cultural beliefs may undermine the early assessment and diagnosis of mental health and neurodevelopmental disorders in African adolescents (Dogra 2015; Garland et al. 2004). In countries like Nigeria, adolescents with intellectual disabilities often go undiagnosed due to inadequate understanding among caregivers and professionals, compounded by the socio‐political climate and inadequate screening tools (Franz et al. 2017; Nwokolo et al. 2024). UNICEF previously projected that 109 million individuals in Nigeria and 1.1 billion across Africa will be under 18 years old by 2100 (UNICEF 2014). Neglecting adolescent developmental needs poses significant challenges (Kieling et al. 2011; Maxey and Beckert 2017; Erskine et al. 2017). The WHO (2014) has proposed strategies, including policy development and intersectoral collaboration, to address this issue. Given the high comorbidity of various disorders in this age group (Jozefiak et al. 2016; Munir 2016; Uzun Cıcek et al. 2020), screening for intellectual disabilities should be integrated into these initiatives.

A diagnosis of intellectual disability requires meeting criteria in three domains: deficits in intellectual functioning, deficits in adaptive skills and onset during the developmental period, according to the ICD‐11 (WHO 2020). Comprehensive intelligence tests can be time‐consuming, require training and are costly, necessitating the use of efficient screening tools for early identification (Franz et al. 2017; Matson et al. 2011). In African countries such as Nigeria, barriers to assessment include a lack of validated screening tools and low awareness among parents and professionals (Franz et al. 2017; Nwokolo et al. 2024).

To explore available time‐ and cost‐efficient screening tools for identifying intellectual disabilities among Nigerian adolescents, Nwokolo et al. (2024) conducted a systematic review of such tools, examining their cultural appropriateness and psychometric properties. The following six tools were identified: the Hayes Ability Screening Index (HASI), the Learning Disability Screening Questionnaire (LDSQ), the Child and Adolescent Intellectual Disability Screening Questionnaire (CAIDS‐Q), the Slosson Intelligence Test (SIT), the Screener for Intelligence and Learning Disabilities (SCIL) and the Quick Test (QT). After assessing the evidence on these tools, two of them (the SCIL and the CAIDS‐Q) were chosen for further review by a focus group of Nigerian experts (Nwokolo et al. 2023). The experts examined the face and content validity of both tools. Following this review, the SCIL was selected for its practical items (Nwokolo et al. 2023). The SCIL (Nijman et al. 2018) is a 14‐item, time‐ and cost‐efficient screening tool that identifies individuals with intellectual disabilities or a borderline level of general intellectual functioning, requiring minimal training for administration. The adolescent version of the SCIL was originally written in Dutch and translated into English, with minor modifications made to enhance cultural appropriateness for Nigerian adolescents (Nwokolo et al. 2023).

To evaluate the adapted SCIL in Nigeria, 209 adolescents and young adults were invited to complete the SCIL alongside assessments of adaptive behaviour and general intellectual functioning. This study aimed to (a) examine the component structure of the SCIL and reduce items as required, (b) examine the internal consistency, discriminative and convergent validity of the SCIL, (c) derive an appropriate cutoff score based upon sensitivity and specificity and (d) derive the positive and negative predictive values.

## 1 Methods

### 1.1 Design

A between‐groups design was used with two groups of participants: (1) adolescents and young people thought to have intellectual disabilities and (2) those thought not to have intellectual disabilities.

### 1.2 Participants

The study was conducted across three cosmopolitan, multiethnic geopolitical zones in Nigeria: Enugu (predominantly Igbo), Abuja (the capital) and Lagos (predominantly Yoruba). Initially, 245 adolescents were invited to participate, but 35 either declined or did not respond to further contact attempts, and one participant did not complete the SCIL. Consequently, 209 adolescents (M_age_ = 15.83 years; Mdn_age_ = 15.28 years; SD = 3.62; Min: 10.90 years; Max: 26.53 years; 41% female and 59% male) took part. The age distribution was categorised as follows: 11–13‐year‐olds (*n* = 76; 36.4%), 14–15‐year‐olds (*n* = 42; 20.1%), 16–17‐year‐olds (*n* = 51; 24.4%) and 18 years and above (*n* = 40; 19.1%).

WISC‐V, WAIS‐III and VABS‐3 were used to confirm the presence or not of intellectual disability, according to diagnostic criteria. Participants suspected of having an intellectual disability were selected if they were (a) aged between 11 and 26 years, (b) identified by a medical doctor as possibly having an intellectual disability and/or (c) attended a mental health centre, a special school or a day centre for individuals with intellectual disabilities. Those believed not to have an intellectual disability were recruited from community organisations, places of worship and through public advertisements, ensuring both groups were included. Participants were classified as having an intellectual disabilitiy or not based on diagnostic criteria using the VABS, WAIS‐III or WISC‐V after recruitment.

### 1.3 Procedure

Participants completed the SCIL and either the Wechsler Adult Intelligence Scale, 3rd Edition (WAIS‐III) or the Wechsler Intelligence Scale for Children, 5th Edition (WISC‐V), depending on age. The Vineland Adaptive Behaviour Scales, 3rd Edition (VABS‐3) were completed with a knowledgeable parent or caregiver. A psychologist administered the WAIS‐III and the WISC‐V, while the VABS‐3 and SCIL were administered by a qualified autism service practitioner supervisor (QASP‐S) trained by the first author (E.N.) on the assessments.

#### 1.3.1 Measures

##### 1.3.1.1 Screener for Intelligence and Learning Disabilities

The Screener for Intelligence and Learning Disabilities (SCIL) (Nijman et al. 2018; Geijsen et al. 2018) is a 14‐item questionnaire for screening intellectual disabilities, taking 10–15 min to complete, scored on a 3‐point Likert scale (0–2 points) with 0 = incorrect or no response, 1 = some correct response and 2 = correct response and a maximum score of 28. Originally developed for Dutch adolescents, it was translated and adapted for use in Nigeria (Nwokolo et al. 2023). The SCIL includes various brief questions, short reading and spelling exercises and a question about the meaning of certain sayings. The original SCIL consists of the following four factors: education; social contacts; arithmetic, reading and writing abilities; and language comprehension and behaviour (Kaal et al. 2013; Geijsen et al. 2018).

##### 1.3.1.2 Wechsler Intelligence Scale for Children, Fifth Edition

The Wechsler Intelligence Scale for Children, Fifth Edition (WISC‐V) (Wechsler 2014) is an intelligence test for children aged 6–16, taking 45–65 min to administer and generating a Full‐Scale IQ (FSIQ) from seven primary subtests. The WISC‐V is scored by converting raw scores into age‐normed scaled scores and composite indices.

##### 1.3.1.3 Wechsler Adult Intelligence Scale, Third Edition

The Wechsler Adult Intelligence Scale, Third Edition (WAIS‐III) (Wechsler 1997) is standardised for older adolescents and adults and used to calculate FSIQ for participants older than 16 years and takes about 60 min to administer. The WAIS‐III is scored by converting raw scores into age‐normed scaled scores and composite indices.

##### 1.3.1.4 Vineland Adaptive Behaviour Scales, Third Edition

The Vineland Adaptive Behaviour Scales, Third Edition (VABS‐3) is a standardised semistructured interview to assess adaptive behaviour, completed with an individual, their carer/parent or a teacher. The carer/parent domain form was used as it is recommended for research purposes (Pepperdine and McCrimmon 2018; Sparrow et al. 2016). The VABS converts raw scores into age‐normed standard scores, percentile ranks and adaptive levels across key domains.

### 1.4 Ethical Opinion

A favourable ethical opinion was obtained from the Tizard Centre, University of Kent, Ethics Committee, the National Health Research Ethics Committee (NHREC; NHREC/01/01/2007‐16/09/2019) and the Federal Neuro‐Psychiatric Hospital, Yaba, Lagos, Nigeria (FNPH/HREC/20/09). Participants received written information about the study, including easier‐to‐read versions. Those under 18 years gave assent and parental consent was obtained, and for those aged 18 and over, they consented for themselves. Participants were allowed breaks as needed during testing.

### 1.5 Data Analysis

#### 1.5.1 Overview

The analysis was conducted using IBM SPSS Version 26. Principal component analysis (PCA) with Promax rotation and Kaiser normalization was used to examine the component structure of the SCIL, following previous analyses of the Dutch version (Geijsen et al. 2018). Items that correlated, *r* = 0.30, with at least one other item were initially retained. Then, those with communalities of at least 0.30 (Floyd and Widaman 1995) were retained, followed by those that had KMO of at least 0.60. Components with eigenvalues greater than 1 were retained.

Following this, discriminative and convergent validity and internal consistency were examined. Convergent validity was assessed by examining the correlation between SCIL scores with Full‐Scale IQ and the VABS‐3. Receiver operating characteristic (ROC) analyses were used to calculate the area under the curve (AUC), alongside sensitivity and specificity, to establish optimal cutoffs. Internal consistency was calculated using Cronbach's *α*, and positive (PPV) and negative predictive values (NPV) calculated.

#### 1.5.2 IQ and VABS Cutoffs for Intellectual Disabilities

Participants were classified as having intellectual disability using ICD‐11 criteria (WHO 2020), which require general intellectual functioning and adaptive behaviour at least two standard deviations below the mean. However, evidence indicates that African populations score approximately one standard deviation lower on Western IQ tests compared to Western norms (Wicherts et al. 2010; Ani and Grantham‐McGregor 1998; Nenty and Dinero 1981; Ashem and Janes 1978; Nwanze and Okeowo 1980; Fahrmeier 1975; Shuttleworth‐Edwards et al. 2013). The validity and cultural appropriateness of these tests in Africa continues to be debated (Kamin 2006; Rushton and Jensen 2006; Bakare et al. 2009; Shuttleworth‐Edwards et al. 2004; Shuttleworth‐Edwards 2012). Factors such as environment, culture, exposure to Western influences and quality of education influence intellectual functioning (Bakare et al. 2009; Shuttleworth‐Edwards et al. 2004, 2013). As Bakare (1972) noted, it is “illogical to use scores on such tests to infer innate ability in people from non‐Western cultures” (362). Warne (2023) further cautioned against direct comparisons of FSIQ scores across Western and developing contexts without establishing measurement invariance. Wicherts et al. (2010) systematically reviewed African performance on Western IQ tests, including the WAIS, WISC, K‐ABC and DAM, reporting an average IQ of approximately 82 relative to UK norms. Similarly, Pienaar et al. (2017) provided preliminary normative guidelines for WAIS‐III and IV among Xhosa‐speaking South African adults, suggesting adjustments of 20–25 points below US norms, with a mean FSIQ of 76.55 (SD = 8.29). These findings underscore the need for caution when interpreting Western IQ scores in African contexts and highlight the importance of cultural and contextual considerations in classification.

Regarding the Vineland Adaptive Behaviour Scales (VABS), several studies compared the VABS to adaptive behaviour measures developed in African countries (Du Toit et al. 2021a, b; De Beer et al. 2020; Allen et al. 2014). However, actual African VABS data were not reported (De Beer et al. 2020; Du Toit et al. 2021a, 2021b; Allen et al. 2014). Douglas (2017) found that the VABS could identify intellectual disabilities in South African sexual abuse victims under 22 years but did not report actual data, making it difficult to examine the VABS performance. Considering the issues and associated cultural sensitivity, we used two different IQ cutoffs for identifying participants with an intellectual disability and completed our analysis four times:

1 Initially, we used the established, **unadjusted**, criterion of FSIQ < 70 (i.e., 2 SD below 100) with the VABS‐3 cutoff of < 70 using Western normative data.
2 Secondly, the **FSIQ cutoff was adjusted** based on the work of Wicherts et al. (2010) to FSIQ score < 52 (i.e., 2 SD below mean Full‐Scale IQ = 82) with the VABS‐3 cutoff of < 70.

As previously mentioned, as far as we could ascertain, the VABS‐3 had not been normed in Nigeria, nor in Africa. Therefore, to determine the effect of excluding the VABS‐3 composite scores, the analysis was repeated excluding the VABS:

3 Using the unadjusted FSIQ < 70 only
4 The adjusted FSIQ < 52 scores only

#### 1.5.3 Unattainable Data

Thirty participants could not complete the WISC‐V, and 7 were unable to complete the WAIS‐III, possibly due to the degree of intellectual disability, with one unable to complete the SCIL. One parent could not finish the VABS‐3. This resulted in 37 participants excluded from analyses involving the Weschler scales, 1 from the VABS Composite scores analysis and 1 from the SCIL analysis.

## 2 Results

### 2.1 Descriptive Statistics

SCIL scores for the whole group ranged from 0 to 28 points (M = 14.01; Mdn = 16.00; SD = 9.4). SCIL scores did not differ between the sexes, *t*(207) = −1.818, *p* = 0.07. Descriptive statistics for the SCIL by age group and sex for the whole sample are in Table 1. Seventy (33.49%) participants had no educational qualifications or were in primary school or special education, 52 (24.88%) had junior secondary education, and 87 (41.63%) had qualifications from senior secondary education or higher. There was no significant correlation between age and the SCIL scores, *r*(209) = 0.13, *p* = 0.06. Tables 2 and 3 show the M and SD for VABS‐3 composite scores and FSIQ scores for the whole group. A significant correlation was found between the VABS‐3 composite scores and FSIQ, *r*(173) = 0.74, *p* < 0.001.

**TABLE 1:** Descriptive statistics of SCIL scores by age groups and sex.

Age groups	*n*	M	SD	Range	Min	Max
11–13 years old	76	12.22	9.29	27	0	27
14–15 years old	42	15.07	8.94	26	0	26
16–17 years old	51	14.43	9.28	28	0	28
18 years old and above	40	15.75	9.98	28	0	28
Male	123	14.99	9.42	28	0	28
Female	86	12.61	9.23	28	0	28
Total	209	14.01	9.40	28	0	28

**TABLE 2:** Descriptive statistics of FSIQ scores by age groups calculated using UK normative data.

Age groups	*n*	M	SD	Range	Min	Max
11–13 years old	57	79.04	20.05	85	42	127
14–15 years old	37	72.97	23.51	89	40	129
16–17 years old	43	74.44	18.56	65	40	105
18 years old and above	36	77.19	25.07	93	45	138
Total	173	76.21	21.55	98	40	138

**TABLE 3:** Descriptive statistics of VABS‐3 composite scores by age groups calculated using US normative data.

Age groups	*n*	M	SD	Range	Min	Max
11–13 years old	76	81.57	25.71	104	20	124
14–15 years old	42	78.98	24.03	96	25	121
16–17 years old	51	77.41	21.96	80	32	112
18 years old and above	40	77.18	25.32	76	37	113
Total	209	79.19	24.33	104	20	124

When the initial criterion (FSIQ < 70) for very low intellectual functioning was applied to the whole sample, 68.42% (*n* = 143) fell below this criterion and 31.58% (*n* = 66) fell above this criterion. However, applying the FSIQ < 52 criterion to the whole sample, 29.67% (*n* = 62) fell below, and 70.33% (*n* = 147) fell above it. Considering the VABS‐3 composite scores criterion of < 70, 69.52% (*n* = 146) fell above this criterion, 30% (*n* = 63) fell below, and 0.48% (*n* = 1) was missing.

Using both unadjusted FSIQ (< 70) and VABS‐3 composite scores (< 70) together to determine whether someone had an intellectual disability revealed that 37% of participants met diagnostic criteria (Table 4). When FSIQ was adjusted (< 52) and considered along with VABS‐3 composite scores (< 70), 16% of our participants met diagnostic criteria for an intellectual disability (Table 5). Thirty‐seven participants who could not complete the WISC‐V and WAIS‐III 7 were excluded reducing the sample size to 173.

**TABLE 4:** Descriptive statistics of the WISC‐V and WAIS‐III scores for participants in the two groups (with and without intellectual disabilities) using an unadjusted criterion for identification of intellectual disability FSIQ score < 70 (i.e., 2 SDs below 100; 37 participants were unable to take part in the assessment) and the VABS‐3 composite score < 70.

	With intellectual disabilities					Without intellectual disabilities				
	*n*	Min	Max	Mean	SD	*n*	Min	Max	Mean	SD
Unadjusted WISC‐V FSIQ	41	40	69	53.51	9.88	80	70	129	88.31	14.18
Unadjusted WAIS‐III FSIQ	23	45	69	53.70	8.01	29	71	138	92.79	14.90
Total participants	64	40	69	53.58	9.19	109	70	138	89.51	14.44
VABS composite score	64	36	104	68.42	15.64	109	70	124	96.51	13.50

*Note:* Participants were grouped based on their test scores.

**TABLE 5:** Descriptive statistics of the WISC‐V and WAIS‐III scores for participants in the two groups (with and without intellectual disabilities) using an adjusted criterion for identification of intellectual disability FSIQ score < 52 (i.e., 2 SDs below 82; excluding the 37 were unable to take part in the assessment) and the VABS‐3 composite score < 70.

	With intellectual disabilities					Without intellectual disabilities				
	*n*	Min	Max	Mean	SD	*n*	Min	Max	Mean	SD
Adjusted WISC‐V FSIQ	18	40	51	43.90	3.97	103	52	129	82.23	17.10
Adjusted WAIS‐III FSIQ	9	45	48	45.78	1.09	43	52	138	81.72	20.47
Total participants	27	40	51	45.52	3.39	146	52	138	82.08	18.09
VABS composite score	27	36	86	59.26	15.58	146	70	124	91.08	16.08

*Note:* Participants were grouped based on their test scores.

### 2.2 Principal Component Analysis of the SCIL (PCA)

The correlation matrix showed all SCIL items correlated (*r* > 0.30) with at least one other item, so all 14 were retained. No multicollinearity was detected (*r* < 0.8). KMO was 0.94, with individual KMOs > 0.87, exceeding the 0.5 threshold (Field 2013; Kaiser 1974). Bartlett's test was significant, ꭓ^2^(91) = 1947.49, *p* < 0.001, confirming item correlation. The scree plot (see supplemental material) suggested three factors, but two factors had eigenvalues > 1, explaining 63.56% variance which we labelled as: (1) education, social support and comprehension (items 1–4, 8–10, 12–13) and (2) arithmetic and numbers (items 5–7, 11, 14). Pattern and structure matrices appear in Table 6.

**TABLE 6:** Pattern and structure matrix of the SCIL.

	Pattern matrix		Structure matrix	
	1	2	1	2
Item 1	**0.79**	0.05	**0.83**	0.60
Item 2	**0.83**	0.02	**0.84**	0.59
Item 3	**0.60**	0.24	**0.77**	0.66
Item 4	**0.93**	−0.40	**0.65**	0.25
Item 5	−0.06	**0.68**	0.41	**0.64**
Item 6	−0.27	**0.97**	0.41	**0.78**
Item 7	−0.11	**0.92**	0.54	**0.85**
Item 8	**0.92**	−0.11	**0.84**	0.53
Item 9	**0.38**	0.24	**0.55**	0.50
Item 10	**0.59**	0.31	**0.81**	0.72
Item 11	0.41	**0.49**	0.75	**0.77**
Item 12	**0.47**	0.46	**0.79**	0.79
Item 13	**0.72**	0.25	**0.90**	0.75
Item 14	0.30	**0.53**	0.67	**0.74**

*Note:* Promax Rotation with Kaiser normalization. Component 1: Eigenvalue 7.77 (% of variance = 55.52), *α* = 0.92. Component 2: Eigenvalue 1.13 (% of variance = 8.04), *α* = 0.84.

### 2.3 Internal Consistency

Internal consistency was high, Cronbach's *α* = 94, 95% CI (0.93, 0.95). This was the case for Component 1, Cronbach's *α* = 0.92, 95% CI (0.91, 0.94), while Component 2 was slightly lower, Cronbach's *α* = 0.84, 95% CI (0.80, 0.87).

### 2.4 Convergent Validity

The correlation between the total SCIL score and FSIQ estimated using the WAIS‐III, *r*(52) = 0.86, *p* < 0.001, and the WISC‐V, *r*(121) = 0.79, *p* < 0.001, was high. Combining both versions of the Wechsler scales, there was a significant positive correlation between the total SCIL score and level of general intellectual functioning, *r*(173) = 0.81, *p* < 0.001. Similarly, there was a significant positive correlation between the total SCIL score and VABS‐3 composite score, *r*(209) = 0.84, *p* < 0.001, and across the VABS domains and the SCIL: communication *r*(209) = 0.86, *p* < 0.001, daily living skills *r*(209) = 0.79, *p* < 0.001 and social skills *r*(209) = 0.74, *p* < 0.001.

### 2.5 Sensitivity, Specificity, PPV and NPV Using Unadjusted Cutoffs (FSIQ and VABS‐3 Composite Scores < 70)

At the suggested SCIL cutoff score of 15 (Nijman et al. 2018), the AUC was 0.96, *p* < 0.001, 95% CI (0.94, 0.98), sensitivity = 0.67, specificity = 0.98, PPV = 0.96 and NPV = 0.82. Exploring lower cutoff scores of 10, 11 and 12 improved sensitivity values (0.95, 0.95 and 0.97, respectively), with specificity between 0.83 and 0.87. There was no difference in sensitivity and specificity using the cutoff scores of 13 and 14 versus the suggested 15. The AUC across the age groups using 15 as the cutoff was: 11–13‐year‐olds, AUC = 0.95, *p* < 0.001, 95% CI (0.90, 0.99), *n* = 76, 14–15‐year‐olds, AUC = 0.99, *p* < 0.001, 95% CI (0.97, 1.01), *n* = 42, 16–17‐year‐olds, AUC = 1, *p* < 0.001, 95% CI (1.0, 1.0), *n* = 51, 18 years and above, AUC = 0.94, *p* < 0.001, 95% CI (0.87, 1.02), *n* = 40. The AUC, PPV, NPV, sensitivity and specificity for each cutoff score are in Table 7. The PPV and NPV with unadjusted cutoffs (*FSIQ and VABS‐3 composite scores* < 70) were also examined. At the suggested SCIL cutoff score of 15 (Nijman et al. 2018), the PPV was 0.96, and the NPV was 0.82. A cutoff score of 12 is suggested for the unadjusted FSIQ of < 70.

**TABLE 7:** Sensitivity, specificity, PPV and NPV for the various potential cutoff scores of the SCIL using the unadjusted mean FSIQ and VABS‐3 composite scores.

	SCIL cutoff score < 10					SCIL cutoff score < 11					SCIL cutoff score < 12				
	Total (*n* = 173)	11–13 years (*n* = 57)	14–15 years (*n* = 37)	16–17 years (*n* = 43)	18 years & above (*n* = 36)	Total (*n* = 173)	11–13 years (*n* = 57)	14–15 years (*n* = 37)	16–17 years (*n* = 43)	18 years & above (*n* = 36)	Total (*n* = 173)	11–13 years (*n* = 57)	14–15 years (*n* = 37)	16–17 years (*n* = 43)	18 years & above (*n* = 36)
PPV	0.76	0.58	0.92	0.94	0.86	0.74	0.58	0.86	0.94	0.86	0.71	0.57	0.86	0.89	0.81
NPV	0.98	1.00	1.00	1.00	0.88	0.98	1.00	1.00	1.00	0.88	0.98	1.00	1.00	1.00	0.92
Sensitivity	0.95	1.00	1.00	1.00	0.80	0.95	1.00	1.00	1.00	0.80	0.97	1.00	1.00	1.00	0.87
Specificity	0.87	0.73	0.97	0.97	0.92	0.86	0.71	0.93	0.97	0.92	0.83	0.71	0.87	0.94	0.88
AUC (*p* < 0.001, 95% CI)	0.78	0.81	0.78	0.80	0.72	0.90	0.86	0.97	0.99	0.86	0.90	0.86	0.93	0.97	0.87
Lower	0.71	0.69	0.61	0.66	0.55	0.86	0.77	0.91	0.95	0.73	0.85	0.77	0.86	0.93	0.75
Upper	0.85	0.93	0.967	0.95	0.89	0.95	0.94	1.02	1.02	1.00	0.94	0.94	1.01	1.02	1.00

### 2.6 Sensitivity, Specificity, PPV and NPV Using Adjusted Cutoffs (FSIQ < 52 and VABS‐3 Composite Scores < 70)

When the adjusted FSIQ cutoff of 52 and VABS‐3 composite scores (< 70) with the suggested SCIL cutoff score of 15 (Nijman et al. 2018), sensitivity was 0.60, specificity 1, PPV 1 and NPV 0.78. Specificity exceeded the minimum standard, but sensitivity did not (Glascoe 2005). The AUC was 0.97, *p* < 0.001, 95% CI (0.95, 0.99). With a cutoff of 15, the AUC across the age groups was large: 11–13‐year‐olds, AUC = 0.98, *p* < 0.001, 95% CI (0.95, 1.01), *n* = 76, 14–15‐year‐olds, AUC = 0.99, *p* < 0.001, 95% CI (0.98, 1.01), *n* = 42, 16–17‐year‐olds, AUC = 0.97, *p* < 0.001, 95% CI (0.92, 1.01), *n* = 51, 18 years and above, AUC = 0.96, *p* < 0.001, 95% CI (0.90, 1.03), *n* = 40.

Lower cutoffs (10, 11 and 12) were explored by stepwise reduction rather than arbitrarily. A cutoff of 10 gave the best result: sensitivity 0.66, specificity 1. No difference was found for a cutoff score of 13 versus 15, so 13 was not explored further. AUCs ranged from 0.84 to 0.99, indicating good discriminative validity. For adjusted cutoff (*FSIQ* < 52) *and VABS‐3 composite scores < 70*: an SCIL cutoff of 10: PPV = 1, NPV = 0.83, cutoff of 11: PPV = 1, NPV = 0.81 and a cutoff of 12: PPV = 1, NPV = 0.78. The SCIL correctly identified those with and without intellectual disabilities. The most suitable cutoff for the adjusted FSIQ was determined to be 10.

Table 8 shows the AUC, PPV, NPV, sensitivity and specificity for each cutoff score; bold figures indicate measurement properties at the applicable cutoff scores.

**TABLE 8:** Sensitivity, specificity, PPV and NPV for the various potential cutoff scores of the SCIL using the adjusted mean FSIQ and VABS‐3 composite scores.

	SCIL cutoff score < 10					SCIL cutoff score < 11					SCIL cutoff score < 12				
	Total (*n* = 173)	11–13 years (*n* = 57)	14–15 years (*n* = 37)	16–17 years (*n* = 43)	18 years & above (*n* = 36)	Total (*n* = 173)	11–13 years (*n* = 57)	14–15 years (*n* = 37)	16–17 years (*n* = 43)	18 years & above (*n* = 36)	Total (*n* = 173)	11–13 years (*n* = 57)	14–15 years (*n* = 37)	16–17 years (*n* = 43)	18 years & above (*n* = 36)
PPV	**1.00**	**1.00**	**1.00**	1.00	1.00	1.00	1.00	1.00	**1.00**	**1.00**	1.00	1.00	1.00	1.00	1.00
NPV	**0.83**	**0.70**	**0.97**	0.85	0.90	0.81	0.68	0.93	**0.85**	**0.90**	0.78	0.68	0.87	0.83	0.83
Sensitivity	**0.66**	**0.53**	**0.92**	0.65	0.79	0.65	0.51	0.86	**0.65**	**0.79**	0.61	0.51	0.75	0.61	0.69
Specificity	**1.00**	**1.00**	**1.00**	1.00	1.00	1.00	1.00	1.00	**1.00**	**1.00**	1.00	1.00	1.00	1.00	1.00
AUC (*p* < 0.001, 95% CI)	0.91	0.85	0.98	0.93	0.95	0.91	0.84	0.97	0.93	0.95	0.89	0.84	0.93	0.91	0.91
Lower	0.88	0.77	0.95	0.85	0.88	0.87	0.78	0.91	0.85	0.88	0.85	0.78	0.86	0.84	0.83
Upper	0.95	0.93	1.02	1.00	1.02	0.95	0.93	1.02	1.00	1.02	0.93	0.93	1.01	0.99	1.00

*Note:* Figures in bold font indicate applicable properties at each cutoff score for the entire population and per age group.

### 2.7 The Effect of Excluding the VABS‐3 Composite Scores

Due to the absence of African VABS‐3 validation data, participants were reassigned to groups with or without intellectual disabilities using only unadjusted and adjusted FSIQ scores, excluding VABS‐3 scores. For unadjusted FSIQ < 70 and SCIL cutoff of 15, total population sensitivity was 0.73, specificity 0.94, PPV 0.89, NPV 0.86 and AUC 0.84. At a cutoff of 10, sensitivity dropped to 0.67, specificity rose to 0.98, PPV 0.96, NPV 0.84 and AUC 0.83. Using adjusted FSIQ < 52, a cutoff of 15 yielded sensitivity 0.93, specificity 0.81, PPV 0.47, NPV 0.98 and AUC 0.87; at 10, sensitivity was 0.89, specificity 0.86, PPV 0.53, NPV 0.98 and AUC 0.87. These results showed only minor differences compared to those using both FSIQ thresholds and VABS‐3 composite score < 70 (Table 9).

**TABLE 9:** Summary of the sensitivity, specificity, PPV and NPV for the various potential cutoff scores of the SCIL using the unadjusted and adjusted mean FSIQ only or with the VABS‐3 composite scores.

	FSIQ < 70 only		FSIQ < 70 with VABS‐3		FSIQ < 52 Only		FSIQ < 52 with VABS‐3	
	Cutoff < 10	Cutoff < 15	Cutoff < 10	Cutoff < 15	Cutoff < 10	Cutoff < 15	Cutoff < 10	Cutoff < 15
PPV	**0.96**	0.89	0.76	**0.96**	**0.53**	0.47	**1.00**	0.94
NPV	0.84	**0.86**	**0.98**	0.82	0.98	0.98	0.83	**0.87**
Sensitivity	0.67	**0.73**	**0.95**	0.67	0.89	**0.93**	**0.66**	0.50
Specificity	**0.98**	0.94	0.87	**0.98**	**0.86**	0.81	1.00	1.00
AUC	0.83	**0.84**	0.78	**0.96**	0.87	0.87	0.91	**0.96**

*Note:* Figures in bold font represent the best value at each cutoff score.

## 3 Discussion

The aims of this study were to examine the component structure, internal consistency, discriminative and convergent validity, the likely appropriate cutoff score based on sensitivity and specificity, as well as the positive and negative predictive values of the SCIL. Early identification of intellectual disabilities is essential for significant progress in intervention, educational support and policy (Luckasson and Schalock 2013; Schalock and Luckasson 2013), but many adolescents in Nigeria have gone undiagnosed in early childhood (Franz et al. 2017).

### 3.1 Component Structure

An analysis of the component structure led to the retention of all 14 items, resulting in two components: (1) education, social support and comprehension and (2) arithmetic and numbers. This contrasts with Geijsen et al. (2018), who retained four components using PCA with Dutch participants aged between 18 and 63 years in police custody. The difference may be due to sampling environment, sample characteristics and/or the version of the SCIL used.

### 3.2 Internal Consistency and Convergent Validity

The internal consistency was excellent. Convergent validity was measured by the relationship between two related constructs: the relationship between the SCIL scores, FSIQ and VABS‐3 composite scores. A positive relationship was found between FSIQ, the VABS‐3 composite and SCIL scores.

### 3.3 Sensitivity, Specificity, Positive Predictive Value and Negative Predictive Value With Unadjusted Cutoffs (FSIQ and VABS‐3 Composite Scores < 70)

ROC analyses were conducted separately for the entire sample and each age group. At the suggested cutoff of 15 (Nijman et al. 2018), sensitivity did not meet the minimum criteria of 70%, while specificity did (Glascoe 2005). At the suggested cutoff of 15, about 67% of participants were designated as having a possible intellectual disability. Lowering the cutoff to 10, 11 and 12 improved the results. The resultant low sensitivity at FSIQ < 70 and VABS‐3 composite score < 70 for the SCIL is possibly due to the use of the Weschler UK standardisation data in the absence of Nigerian standardisation data. Psychometric assessments in cross‐cultural environments are problematic, considering the role that culture, beliefs, language, ethnicity, exposure and quality of education play in test‐taking.

### 3.4 Sensitivity, Specificity, Positive Predictive Value and Negative Predictive Value With Adjusted Cutoffs (FSIQ < 52 and VABS‐3 Composite Scores < 70)

ROC analyses were conducted for the entire sample and by age group using the adjusted criteria (FSIQ < 52 and VABS‐3 composite < 70). Across all analyses, specificity, PPV and NPV met the 70% threshold recommended for screening tools (Glascoe 2005), while sensitivity remained acceptable at 66%. For the overall sample, an SCIL cutoff score of 10 was appropriate, with slight variations (10 or 11) across age groups. These differences reflect the balance between sensitivity and PPV and specificity and NPV in screening and clinical contexts (Trevethan 2017; Akobeng 2007). Unlike sensitivity and specificity, PPV and NPV depend on prevalence and the population studied, influencing classification outcomes. Concerns raised by Nijman et al. (2018) regarding the use of the SCIL with younger populations (12–13 years) were addressed, as findings showed the tool could identify intellectual disabilities in this group. Given developmental variability, age‐specific cutoffs are beneficial, although all were close to the overall cutoff of 10 derived from adjusted FSIQ.

Regarding the sensitivity and specificity of the SCIL, we also examined the PPV and negative NPV to evaluate its effectiveness in identifying individuals with or without the condition. Trevethan (2017) noted that while sensitivity and specificity reflect a test's ability to classify a reference group known to have or not have a condition, PPV and NPV are the probabilities that a test will correctly classify those with and without the condition when their status is unknown. Our results indicate that the SCIL is suitable for screening intellectual disabilities in Nigerian adolescents, with adequate sensitivity, specificity, PPV and NPV, even for those under 14, addressing concerns raised by Nijman et al. (2018).

### 3.5 The Effect of Excluding the VABS‐3 composite Scores

Although the VABS‐3 has not been normed for use in Nigeria or across Africa, significantly below average adaptive skills are an essential part of ICD‐11 diagnostic criteria for intellectual disability. Uncertainty about the cultural appropriateness of the VABS‐3 remains. In a social context, expected behaviours are defined by others and are therefore highly culturally dependent. For example, the ability to complete a task may be hindered by limitations set by others (domestic staff, skills not taught, or a custodial environment where others perform tasks). To better understand these issues, the VABS‐3 was excluded, and the analysis was rerun. Excluding the VABS‐3 led to minor changes in the psychometric values of the SCIL. At the suggested cutoff score of 15, using the unadjusted FSIQ < 70 alone, the SCIL showed better sensitivity (0.73) than when participants were classified using both FSIQ and VABS‐3 < 70 (0.67). However, at the lower cutoff score of 10, sensitivity improved (0.95). When adjusted FSIQ was < 52, the specificity of the SCIL remained acceptable for both cutoff scores of 10 and 15, although sensitivity was lower, at 0.66 and 0.50 respectively. Additionally, at FSIQ < 52, the PPV did not reach the recommended threshold of 0.70 for either cutoff. Including VABS‐3 in the classification introduced a stricter criterion requiring both cognitive and adaptive deficits, thereby reducing the number of individuals identified and lowering SCIL's sensitivity.

### 3.6 Limitations

This study has several limitations. First, the SCIL was administered to a sample mainly from large urban cities in Nigeria, potentially introducing bias as this is not representative of the entire population. Second, the sample size was reduced by 37 participants who could not complete the assessment; while they likely had intellectual disabilities, their exclusion limits the FSIQ analysis. Despite this, the remaining sample size was sufficient for our analyses. Third, the test–retest reliability of the SCIL was not evaluated. Fourth, the Wechsler scales have limited use, and the VABS‐3 has not been validated for Nigeria, thus creating uncertainty around the appropriate cutoff scores for identifying intellectual disabilities.

The degree of uncertainty regarding the cultural sensitivity of the VABS‐3 is currently unknown; however, in the absence of an alternative measure for adaptive behaviour and considering the observations from Tan et al. (2014) and other VABS studies previously mentioned, we utilised the VABS‐3, retaining existing cutoffs. Further VABS‐3 validation work in Nigeria and Africa is needed. Lastly, the adjustments to the FSIQ used to identify participants with intellectual disabilities are highly sensitive in nature, which we wish to recognise. However, as previously mentioned, there are studies to support the adjustments, and the decision was made in the absence of Nigerian standardisation data. Despite these limitations, our findings indicated that the SCIL is a likely valid screening tool for intellectual disability in Nigerian adolescents.

### 3.7 Conclusions

Our findings indicated that the English version of the adolescent SCIL has good construct, convergent and discriminative validity. The SCIL can offer a valuable means of identifying adolescents likely to have an intellectual disability at an earlier stage so that they can be referred for diagnosis before initiating interventions (Franz et al. 2017) or providing targeted support (Kieling et al. 2011). As a simple and quick screening tool, further research is recommended to utilise the English version of the adolescent SCIL in more Nigerian cities and other English‐speaking African populations.

## Funding

The authors have nothing to report.

## Conflicts of Interest

Xavier Moonen is one of the developers of the Dutch version of the SCIL. He receives an annual financial contribution from the publisher for, among other things, further research. The other authors declare no conflicts of interest.

## Supporting information




**Data S1:** Supporting Information.

## Data Availability

The data that support the findings of this study are available from the corresponding author upon reasonable request.
