# Supplementary material for: Preliminary Testing of the English Version of the Adolescent Screener for Intelligence and Learning Disabilities (SCIL) Among Adolescents in Nigeria
Source: J Intellect Disabil Res. 2026 Jul 2;70(10):1033–42. doi: 10.1111/jir.70136 (PMC13553317; doi:10.1111/jir.70136)
Supplement: Supplementary file 1 [file JIR-70-1033-s001.docx]

**Endorsement patterns of the SCIL items**

|  | | | |
| --- | --- | --- | --- |
|  | Classification | |  |
| SCIL_ITEM_1 | No ID | ID | Total |
| 0 | 17 | 49 | 66 |
| 1 | 0 | 1 | 1 |
| 2 | 129 | 13 | 142 |
| Total | 146 | 63 | 209 |
|  | | | |
|  | | | |

| *Chi-Squared Tests* | | | |
| --- | --- | --- | --- |
|  | Value | df | p |
| Χ² | 92.98 | 2 | < .001 |
| N | 209 |  |  |
|  | | | |
|  | | | |

|  | | | |
| --- | --- | --- | --- |
|  | Classification | |  |
| SCIL_ITEM_2 | No ID | ID | Total |
| 0 | 15 | 55 | 70 |
| 1 | 48 | 4 | 52 |
| 2 | 83 | 4 | 87 |
| Total | 146 | 63 | 209 |
|  | | | |
|  | | | |

| *Chi-Squared Tests* | | | |
| --- | --- | --- | --- |
|  | Value | df | p |
| Χ² | 117.4 | 2 | < .001 |
| N | 209 |  |  |
|  | | | |
|  | | | |

|  | | | |
| --- | --- | --- | --- |
|  | Classification | |  |
| SCIL_ITEM_3 | No ID | ID | Total |
| 0 | 40 | 61 | 101 |
| 2 | 106 | 2 | 108 |
| Total | 146 | 63 | 209 |
|  | | | |
|  | | | |

| *Chi-Squared Tests* | | | |
| --- | --- | --- | --- |
|  | Value | df | p |
| Χ² | 84.95 | 1 | < .001 |
| N | 209 |  |  |
|  | | | |

|  | | | | |
| --- | --- | --- | --- | --- |
|  | | Classification | |  |
| SCIL_ITEM_4 | | No ID | ID | Total |
| 0 | | 3 | 26 | 29 |
| 2 | | 143 | 37 | 180 |
| Total | | 146 | 63 | 209 |
|  | | | | |
|  | | | | |
| *Chi-Squared Tests* | | | |  |
|  | Value | df | p |  |
| Χ² | 56.63 | 1 | < .001 |  |
| N | 209 |  |  |  |
|  | | | |  |

|  | | | |
| --- | --- | --- | --- |
|  | Classification | |  |
| SCIL_ITEM_5 | No ID | ID | Total |
| 0 | 104 | 63 | 167 |
| 2 | 42 | 0 | 42 |
| Total | 146 | 63 | 209 |
|  | | | |
|  | | | |

| *Chi-Squared Tests* | | | |
| --- | --- | --- | --- |
|  | Value | df | p |
| Χ² | 22.68 | 1 | < .001 |
| N | 209 |  |  |
|  | | | |

|  | | | |
| --- | --- | --- | --- |
|  | Classification | |  |
| SCIL_ITEM_6 | No ID | ID | Total |
| 0 | 97 | 63 | 160 |
| 2 | 49 | 0 | 49 |
| Total | 146 | 63 | 209 |
|  | | | |
|  | | | |

| *Chi-Squared Tests* | | | |
| --- | --- | --- | --- |
|  | Value | df | p |
| Χ² | 27.62 | 1 | < .001 |
| N | 209 |  |  |
|  | | | |

|  | | | |
| --- | --- | --- | --- |
|  | Classification | |  |
| SCIL_ITEM_7 | No ID | ID | Total |
| 0 | 75 | 62 | 137 |
| 2 | 71 | 1 | 72 |
| Total | 146 | 63 | 209 |
|  | | | |
|  | | | |

| *Chi-Squared Tests* | | | |
| --- | --- | --- | --- |
|  | Value | df | p |
| Χ² | 43.13 | 1 | < .001 |
| N | 209 |  |  |
|  | | | |

|  | | | | |
| --- | --- | --- | --- | --- |
|  | | Classification | |  |
| SCIL_ITEM_8 | | No ID | ID | Total |
| 0 | | 9 | 47 | 56 |
| 1 | | 1 | 2 | 3 |
| 2 | | 136 | 14 | 150 |
| Total | | 146 | 63 | 209 |
|  | | | | |
|  | | | | |
|  | | | | |
| *Chi-Squared Tests* | | | |  |
|  | Value | df | p |  |
| Χ² | 109.7 | 2 | < .001 |  |
| N | 209 |  |  |  |
|  | | | |  |
|  | | | |  |

|  | | | | |
| --- | --- | --- | --- | --- |
|  | | Classification | |  |
| SCIL_ITEM_9 | | No ID | ID | Total |
| 0 | | 83 | 59 | 142 |
| 2 | | 63 | 4 | 67 |
| Total | | 146 | 63 | 209 |
|  | | | | |
|  | | | | |
| *Chi-Squared Tests* | | | |  |
|  | Value | df | p |  |
| Χ² | 27.37 | 1 | < .001 |  |
| N | 209 |  |  |  |
|  | | | |  |

|  | | | |
| --- | --- | --- | --- |
|  | Classification | |  |
| SCIL_ITEM_10 | No ID | ID | Total |
| 0 | 36 | 59 | 95 |
| 1 | 2 | 0 | 2 |
| 2 | 108 | 4 | 112 |
| Total | 146 | 63 | 209 |
|  | | | |
|  | | | |
| *Chi-Squared Tests* | | | |
|  | Value | df | p |
| Χ² | 84.51 | 2 | < .001 |
| N | 209 |  |  |
|  | | | |
|  | | | |

|  | | | |
| --- | --- | --- | --- |
|  | Classification | |  |
| SCIL_ITEM_11 | No ID | ID | Total |
| 0 | 22 | 54 | 76 |
| 1 | 40 | 8 | 48 |
| 2 | 83 | 2 | 85 |
| 4 | 1 | 0 | 1 |
| Total | 146 | 64 | 210 |
|  | | | |
|  | | | |

| *Chi-Squared Tests* | | | |
| --- | --- | --- | --- |
|  | Value | df | p |
| Χ² | 95.54 | 3 | < .001 |
| N | 210 |  |  |
|  | | | |
|  | | | |

|  | | | |
| --- | --- | --- | --- |
|  | Classification | |  |
| SCIL_ITEM_12 | No ID | ID | Total |
| 0 | 37 | 60 | 97 |
| 1 | 16 | 2 | 18 |
| 2 | 92 | 2 | 94 |
| 4 | 1 | 0 | 1 |
| Total | 146 | 64 | 210 |
|  | | | |
|  | | | |

| *Chi-Squared Tests* | | | |
| --- | --- | --- | --- |
|  | Value | df | p |
| Χ² | 84.36 | 3 | < .001 |
| N | 210 |  |  |
|  | | | |
|  | | | |

|  | | | |
| --- | --- | --- | --- |
|  | Classification | |  |
| SCIL_ITEM_13 | No ID | ID | Total |
| 0 | 18 | 54 | 72 |
| 1 | 22 | 5 | 27 |
| 2 | 106 | 4 | 110 |
| Total | 146 | 63 | 209 |
|  | | | |
|  | | | |

| *Chi-Squared Tests* | | | |
| --- | --- | --- | --- |
|  | Value | df | p |
| Χ² | 107.2 | 2 | < .001 |
| N | 209 |  |  |
|  | | | |
|  | | | |

|  | | | |
| --- | --- | --- | --- |
|  | Classification | |  |
| SCIL_ITEM_14 | No ID | ID | Total |
| 0 | 54 | 61 | 115 |
| 1 | 13 | 0 | 13 |
| 2 | 79 | 2 | 81 |
| Total | 146 | 63 | 209 |
|  | | | |
|  | | | |

| *Chi-Squared Tests* | | | |
| --- | --- | --- | --- |
|  | Value | df | p |
| Χ² | 63.71 | 2 | < .001 |
| N | 209 |  |  |
|  | | | |
|  | | | |
